# Effects of creatine and β-guanidinopropionic acid and alterations in creatine transporter and creatine kinases expression in acute seizure and chronic epilepsy models

**DOI:** 10.1186/1471-2202-11-141

**Published:** 2010-10-28

**Authors:** Dae Won Kim, Seong-Il Yeo, Hea Jin Ryu, Ji-Eun Kim, Hong-Ki Song, Oh-Shin Kwon, Soo Young Choi, Tae-Cheon Kang

**Affiliations:** 1 Department of Biomedical Sciences, College of Life Science, Hallym University, Chunchon Kangwon-Do 200-702, Republic of Korea; 2 Department of Anatomy and Neurobiology, College of Medicine, Hallym University, Chunchon, Kangwon-Do 200-702, Republic of Korea; 3 Institute of Epilepsy Research, College of Medicine, Hallym University, Chunchon, Kangwon-Do 200-702, Republic of Korea; 4 Department of Neurology, College of Medicine, Hallym University, Chunchon, Kangwon-Do 200-702, Republic of Korea; 5 Department of Biochemistry, College of Natural Science, Kyungpook National University, Taegu 702-702, Republic of Korea

## Abstract

**Background:**

In order to confirm the roles of creatine (Cr) in epilepsy, we investigated the anti-convulsive effects of Cr, creatine transporter (CRT) and creatine kinases (CKs) against chemical-induced acute seizure activity and chronic epileptic seizure activity.

**Results:**

Two hr after pilocarpine (PILO)-seizure induction, ubiquitous mitochondrial CK (uMtCK) immunoreactivity was unaltered as compared to control level. However, brain-type cytoplasm CK (BCK) immunoreactivity was decreased to 70% of control level. CRT immunoreactivity was decreased to 60% of control level. Following Cr or Tat-CK treatment, uMtCK or CRT immunoreactivity was unaffected, while BCK immunoreactivity in Cr treated group was increased to 3.6-fold of control levels. β-Guanidinopropionic acid (GPA, a competitive CRT inhibitor) reduced BCK and CRT expression. In addition, Cr and tat-BCK treatment delayed the beginning of seizure activity after PILO injection. However, GPA treatment induced spontaneous seizure activity without PILO treatment. In chronic epilepsy rats, both uMtCK and CRT immunoreactivities were reduced in the hippocampus. In contrast, BCK immunoreactivity was similar to that observed in control animals. Cr-, GPA and tat-BCK treatment could not change EEG.

**Conclusion:**

Cr/CK circuit may play an important role in sustaining or exacerbating acute seizure activity, but not chronic epileptic discharge.

## Background

Maintenance of energy homeostasis in the brain requires a distinct molecular circuitry which provides tight coupling between energy consumption and production during the performance of sensory, motor and cognitive processes [1,2]. It is generally assumed that most energy required in the nervous system is provided in the form of adenosine triphosphate (ATP) by mitochondria [3]. ATP production by glycolysis in glia [4] and neurons has been recognized as an alternative source of energy [5,6]. Furthermore, local ATP/ADP ratios and proper distribution of metabolic energy are maintained by catalyzed exchange of high-energy phosphoryls between γ-ATP and phosphocreatine (PCr) or β-adenosine diphosphate (β-ADP) [7,8].

The brain is a main target in infants with creatine (Cr)-deficiency syndrome; the patients exhibit delayed psychomotor development, hypotonia, seizure and myelination delay [9-13]. Cr biosynthesis involves tow sequential steps catalyzed by L-arginine:glycine amidiontransferase (AGAT) and S-adenosylmethionine:guanidinoacetate N-methyltransferase (GAMT). Cr is mainly synthesized in the liver and pancreas [14], and continuously transported to the brain via creatine transporter (CRT) across the blood-brain barrier [15]. Cr is also synthesized in the brain [16]. Astrocytes have been shown to synthesize creatine from glycine added to culture media [17]. Phosphate transfer reaction between Cr and PCr is reversibly catalyzed by creatine kinases (CKs) present in the mitochondria and cytoplasm [18]. Four CK isozymes have been identified, ubiquitous mitochondrial CK (uMtCK), sarcomeric mitochondrial CK, brain-type cytoplasm CK (BCK) and muscle-type cytoplasmic CK [14]. In the brain, uMtCK and BCK are expressed. CK reaction (MgATP2^- ^+ Cr ↔ MgADP^- ^+ PCr2^- ^+ H^+^) plays an important role in regulation of PCr tissue level upon physiological stimulation [19]. Thus, CKs have an energy protective role in the brain. Cr feeding increases survival under experimentally stressed, hypoxic/ischemic conditions [20,21]. Furthermore, PCr/Cr loading has been demonstrated by showing beneficial effects of Cr-feeding in children with inborn errors in Cr transport [22].

On the other hand, ATP metabolic rates in cerebral hemisphere, including cerebral blood flow as well as anabolic and aerobic glycolysis increase two to three times during seizures [21]. It has been also reported that seizure decreases total glycogen level to 60%, and increases lactate to more than sevenfold as compared to pre-ictal condition [23]. However, the levels of ATP and glucose did not change [23] and energy homeostasis was maintained [24]. Thus, seizures seem to provoke coupling of CK activity rather than glucose and oxygen consumption. However, the roles of CK in seizure activity are still controversial. Pentylenetetrazol (PTZ)-induced seizure activity increases local cerebral glucose utilization [25], a higher CK rate constant and a high ATP turnover [26]. However, BCK knockout (KO) mice have showed significantly more myoclonic jerks, while slower chemically induced seizure development [27]. Furthermore, Eraković et al. [28] have also reported that lithium-pilocarpine-induced status epilepticus does not influence CK activity. Therefore, the present study was undertaken to evaluate the potential anti-convulsive effects of Cr, creatine transporter and creatine kinases against in chemical-induced acute seizure activity and chronic epileptic seizure activity

## Methods

### Experimental animals and chemicals

This study utilized the progeny of Sprague-Dawley (SD) rats (male, 9 weeks old) obtained from Experimental Animal Center, Hallym University, Chunchon, South Korea. The animals were provided with a commercial diet and water *ad libitum *under controlled temperature, humidity and lighting conditions (22 ± 2°C, 55 ± 5% and a 12:12 light/dark cycle with lights). The procedures involving animals and their care were conducted in conformity with the institutional guidelines and in compliance with international laws and policies (NIH Guide for the Care and Use of Laboratory Animals, NIH Publication No. 80-23, 1996). All reagents were obtained from Sigma-Aldrich (St. Louis, MO), except as noted.

### Drug treatment

Animals were injected intraperitoneally with saline, Cr (200 mg/kg, I.P.) or β-guanidinopropionic acid (GPA, a competitive creatine transporter inhibitor, 400 mg/kg, I.P.) once in everyday at 7 days before experimental procedures [29-32]. The dosage of drugs was chosen based on the preliminary study.

### Construction and purification of Tat-BCK fusion proteins

To generate a cell-permeable expression vector, Tat-BCK, and CK cDNA was subcloned into the pET-15b plasmid that had been reconstructed to contain the Tat peptide. The Tat-BCK expression vector thus formed contained consecutive cDNA sequences encoding BCK, HIV-1 Tat peptide and six histidine residues at the amino-terminus. We also constructed the BCK expression vector to produce control BCK protein without Tat transduction peptides (data not shown). Following the induction of expression, the Tat-BCK fusion proteins were purified. The fusion proteins were expressed in *E. coli *and the clarified cell extracts were loaded onto a Ni^2+^-nitrilotriacetic acid sepharose affinity column. Fusion protein containing fractions was combined and salts were removed using a PD10 column [33].

### Western blot analysis

**Figure 1:**
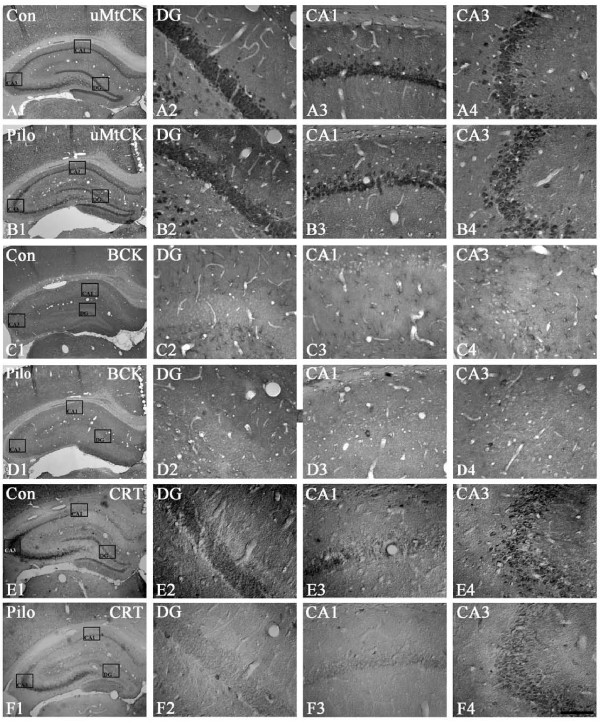
**Changes in uMtCK (A, B), BCK (C, D) and CRT (E, F) immunoreactivity in the rat hippocampus at 2 hr after PILO injection: (A, C, E) saline-treated animal; (B, D, F) PILO-treated animal**. uMtCK immunoreactivity is unaffected by PILO-induce seizure activity. However, both BCK and CRT immunoreactivities are markedly reduced in the rat hippocampus at 2 hr after PILO injection. Rectangles in panels 1 indicate the regions of panels 2 and 3. Bar = 200 (panel 1) and 25 (panels 2-4) μm.

To evaluate efficiency of Tat-BCK transduction, animals were injected intraperitoneally Tat-protein, BCK or Tat-BCK (15 mg/kg, n = 3, respectively). One hr after injection, animals were used in Western blot study. After sacrifice and removal of hippocampi, the tissues were homogenized in 50 mM HEPES (pH 7.4), 0.1 mM EGTA (pH 8.0), 0.2% NP-40, 10 mM EDTA (pH 8.0), 15 mM sodium pyrophosphate, 100 mM h-glycerophosphate, 50 mM NaF, 150 mM NaCl, 2 mM sodium orthovanadate, 1 mM PMSF, and 1 mM DTT. After centrifugation at 15,000 g, the protein concentration was determined in the supernatants by using the Micro BCA protein assay kit with bovine serum albumin as the standard (Pierce Chemical, Rockford, IL, USA). Aliquots containing 50 μg total proteins were boiled in loading buffer containing 150 mM Tris (pH 6.8), 3 mM DTT, 6% SDS, 0.3% bromophenol blue, and 30% glycerol. Then, each aliquot was loaded onto a 12% polyacrylamide gel. After electrophoresis, the gels were transferred onto nitrocellulose transfer membranes (Schleicher and Schuell, Keene, NH, USA). The membrane was blocked in 5% nonfat milk in Tris-buffered saline (TBS; 20 mM Tris, 0.2 M NaCl, pH 7.5) containing 0.05% tween-20 (TBST) for 2 h and was then incubated for 1 h at room temperature with anti-histidine antibody in TBST. After washing, the membrane was incubated for 1 h with a proper secondary antibody conjugated to horseradish peroxidase diluted 1:10000 in TBST. The membrane was incubated with a chemiluminescent substrate and exposed to Hyperfilm ECL (Amersham Biosciences, Piscataway, NJ, USA) [34-36]. As the results, histidine immunoband was detected in the Tat-BCK injected animals (Figure 1B).

### Acute seizure and chronic epilepsy model

Rats were treated with pilocarpine (PILO, 380 mg/kg, i.p.) at 20 min after methylscopolamine (5 mg/kg, i.p.) and were placed in individual observation chambers where seizure activity was scored according to the system of Racine [37]. Animals that entered SE typically did so within 10 to 30 min of the administration of PILO and exhibited continuous seizure activity of between 2 and 5 on the Racine scale. For acute seizure models, animals were anesthetized (urethane, 1.5 g/kg, I.P.) and perfused via the ascending aorta with 200 ml of 4% paraformaldehyde in phosphate buffer (see tissue processing and immunohistochemistry) at 2 hr after PILO injection (N = 5, respectively). For chronic epilepsy model, diazepam (10 mg/kg, i.p.) was administered 2 hrs after onset of SE and repeated, as needed. Following SE, animals were observed 3 - 4 hrs a day in the vivarium for general behavior and occurrence of spontaneous seizures for 4 weeks. The onset of spontaneous seizure occurrence was approximately 3 - 4 weeks after SE (n = 5, respectively). Spontaneous seizures were scored grade 3 or greater on the Racine [37] scale (i.e., forelimb clonus ± rearing ± falling). These behavioral results were consistent with our previous studies [38,39]. Control animals were treated as for experimental animals, but received saline in place of PILO. In addition, 30 min before PILO treatment, diazepam (10 mg/kg, i.p.) was given to some animals. Diazepam pretreatment completely prevented SE. The PILO or saline treatment was performed in the same day. Age matched rats were used as controls. Data from every group of control animals were pooled because there was no difference between groups.

### Electrophysiology

Rats were anesthetized (urethane, 1.5 g/kg, I.P.) and placed in a stereotaxic frame. Holes were drilled through the skull to introduce electrodes. The coordinates (in mm) were as follows. For the recording electrode (to the dentate gyrus): - 3.8 anterior-posterior, 2.5 lateral to bregma, 2.9 depth, at a right angle to the skull surface. For the stimulating electrode (to the angular bundle): 4.2 lateral to lambda, 3.0 depth. Stainless steel electrode (Plastics One Inc, USA) was used for recording. The reference electrode was placed in the posterior cranium over the cerebellum. Signals were recorded with DAM 80 differential amplifier (0.1-3000 Hz bandpass, World Precision Instruments, USA) and data were digitized (20 kHz) and analyzed on MacChart 5 (AD Instruments, Australia). After establishing a stable baseline for at least 30 min, PILO (380 mg/kg, I.P.) were given at 20 min after methylscopolamine (5 mg/kg, i.p.), and observed the latency (n = 5, respectively). Some animals were injected intraperitoneally with tat-BCK or tat-protein (15 mg/kg, I.P., respectively, n = 5, respectively) after baseline recording. The dosage of tat-BCK was chosen based on the preliminary study. Thirty min after administration, PILO (380 mg/kg, I.P.) were given at 20 min after methylscopolamine (5 mg/kg, i.p.), and observed the latency. The latency was determined as seconds from the PILO injection time point to the time point showing the first seizure activity [40]. The same method was applied to chronic epilepsy rats without PILO and methylscopolamine treatments. To analyze changes in EEG power value in chronic epilepsy, root mean square (RMS) value was measured during the baseline and post-tat-BCK treatment measurements.

### Tissue processing and immunohistochemistry

Animals were anesthetized (urethane, 1.5 g/kg, I.P.) and perfused via the ascending aorta with 200 ml of 4% paraformaldehyde in phosphate buffer. Control animals were also perfused with the same method. The brains were removed, postfixed in the same fixative for 4 h, and rinsed in PB-containing 30% sucrose at 4°C for 2 days. Thereafter, the tissues were frozen and sectioned with a cryostat at 30 μm, and consecutive sections were collected in six-well plates containing phosphate buffered saline (PBS). These free-floating sections were first incubated with 10% normal horse serum for 30 min at room temperature. They were then incubated in the rabbit anti-BCK(Santa Cruz, USA, diluted 1:200), uMtCK (Santa Cruz, USA, diluted 1:200) or CRT (Abcam, UK, diluted 1:200) in PBS containing 0.3% triton X-100 and 2% normal chicken serum overnight at room temperature. After washing three times for 10 min with PBS, sections were incubated sequentially, in secondary antibody and ABC complex (Vector, USA), diluted 1:200 in the same solution as the primary antiserum. Between the incubations, the tissues were washed with PBS three times for 10 min each. The sections were visualized with 3,3'-diaminobenzidine (DAB) in 0.1 M Tris buffer and mounted on the gelatin-coated slides. The immunoreactions were observed under the Axioscope microscope (Carl Zeiss, Germany). For double immunofluorescent study, sections were then incubated in a mixture of rabbit anti-BCK(Santa Cruz, USA, diluted 1:100) or CRT (Abcam, UK, diluted 1:100)/mouse anti-GFAP IgG (Chemicon, USA, diluted 1:4 k) in PBS containing 0.3% triton X-100 overnight at room temperature. After washing three times for 10 minutes with PBS, sections were also incubated in a mixture of FITC- and Cy3-conjugated secondary antiserum (1:250, Amersham, USA) for 2 hr at room temperature. The sections were washed three times for 10 min with PBS, and mounted on gelatin-coated slides. For nuclei counterstaining, we used Vectashield mounting medium with DAPI (Vector, USA). All images were captured using an Axiocam HRc camera and Axio Vision 3.1 software. To establish the specificity of the immunostaining, a negative control test was carried out with preimmune serum instead of primary antibody. The negative control resulted in the absence of immunoreactivity in any structures. All experiment procedures in this study were performed under the same circumstance and in parallel.

### Quantification of data and statistical analysis

Sections (5 sections per each animal) were viewed through a microscope connected via a CCD camera to a PC monitor. For quantification of immunohistochemical data, images of each section (including the molecular layer and the granule cell layer of the DG) on the monitor were captured. The mean gray value and its standard deviation were obtained from the selected images in Adobe Photoshop 8.0. Each image was normalized by assessing the mean gray value. After regions (CA1, 3 pyramidal cell layers and dentate granule cell layer) were outlined, 10 areas per rat (300 μm^2^/area) were then selected from the hippocampus, and this area was used for analysis. The images were analyzed by converting all immunolabeled elements that fall within a threshold range into pure black pixels, and the rest of the image is converted into pure white pixels. Then percentage of pure black and white pixels was calculated. After this, the integral signal intensity values were divided by the corresponding area (300 μm^2^), and the average of optical density was calculated. Values of background staining were obtained from the corpus callosum. Optical density values obtaining from immunohistochemistry were corrected by subtracting the average values of background noise obtained from five image inputs. The optical density was then standardized by setting the threshold levels. Intensity measurements were represented as the mean number of a 256 gray scale (using NIH Image 1.59 software). All data were analyzed using Student t-test or one-way analysis of variance (ANOVA) test to determine statistical significance. Bonferroni's test was used for post hoc comparisons. The statistical tests relied on comparison of each time point to a pool of all time points from saline-treated controls (because there was no difference between these control time points). All values were expressed as mean ± SEM. A P-value below either 0.01 or 0.05 was considered statistically significant [34-36].

## Results

### The effects of PILO-induced seizure activity on CK and CRT expressions in the rat hippocampus

In saline-treated animals, uMtCK immunoreactivity was observed in CA1-3 pyramidal cells, dentate granule cells and hilar neurons (Figures 1A). One day after SE, uMtCK immunoreactivity was unaltered as compared to control level (Figures 1B). In saline-treated animals, BCK immunoreactivity was noticeably absent in pyramidal cells and dentate granule cells, while its immunoreactivity was obviously detected in some glial component and in a small subpopulation of hilar neurons (Figures 1C). Double immunofluorescent study revealed that BCK positive glial component was astrocytes (data not shown). One day after SE, BCK immunoreactivity in astrocytes was decreased to 70% of control level (P < 0.01 vs. control, Figures 1D). In saline-treated animals, CRT immunoreactivity was mainly observed in dentate granule cells, hilar neurons and CA3 pyramidal cells (Figures 1E). One day after SE, CRT immunoreactivity in dentate granule cells and CA3 pyramidal cells was decreased to 76 and 52% of control level, respectively (P < 0.05 vs. control, Figures 1F).

### The effects of Cr, GPA and tat-BCK on CK and CRT expressions in the rat hippocampus

**Figure 2:**
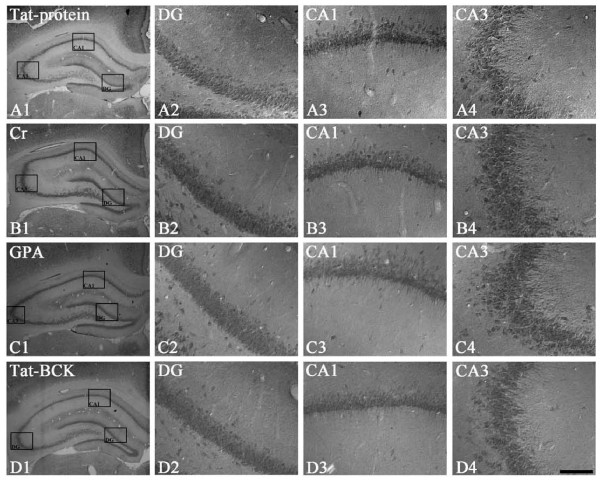
**The effect of Tat-protein (A), Cr (B), GPA (C) or Tat-BCK (D) treatment on uMtCK immunoreactivity in the rat hippocampus**. uMtCK immunoreactivity is unaltered after Tat-protein, Cr, GPA or Tat-BCK treatment. Rectangles in panels 1 indicate the regions of panels 2 and 3. Bar = 200 (panel 1) and 25 (panels 2-4) μm.

**Figure 3:**
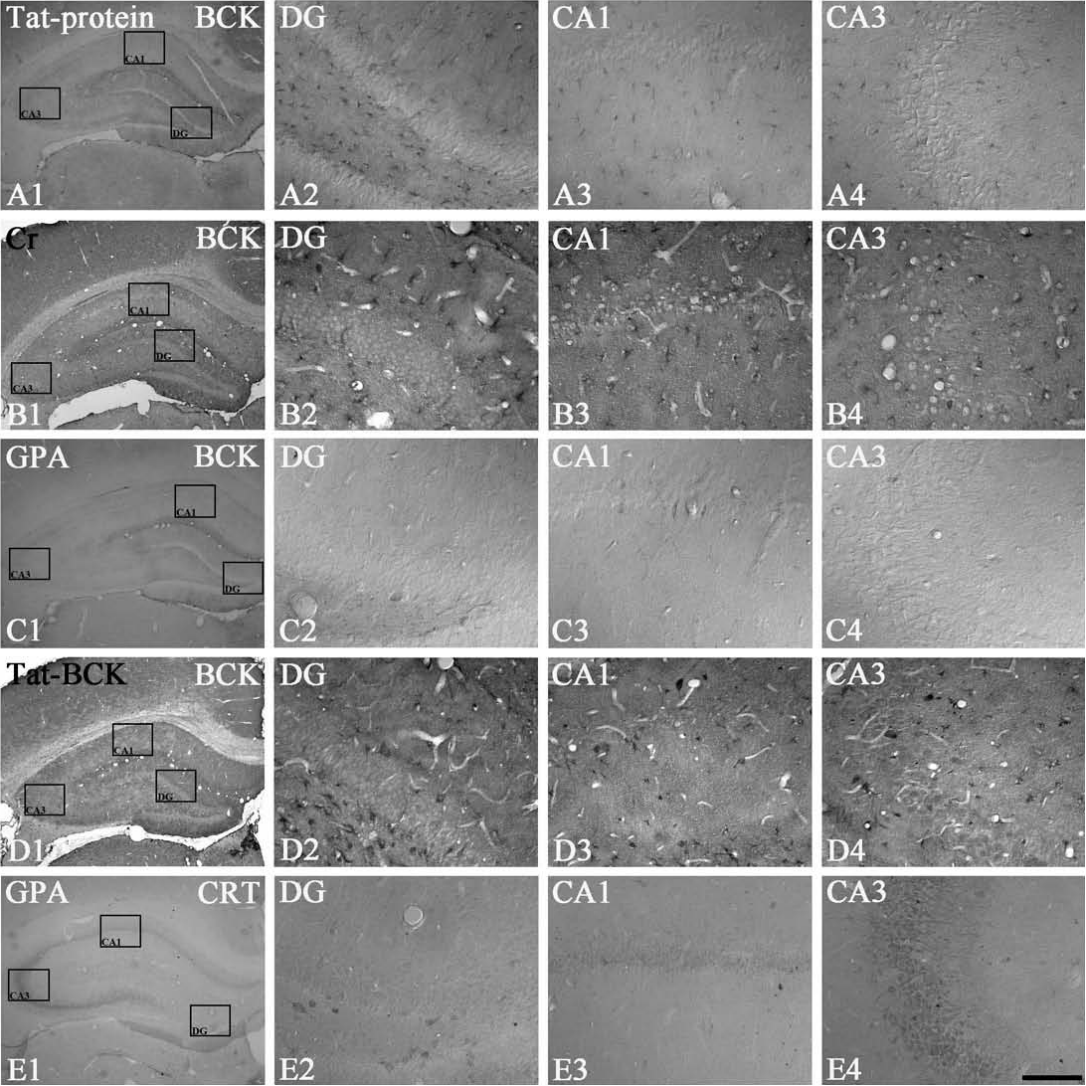
**The effect of Tat-protein (A), Cr (B), GPA (C, E) or Tat-BCK (D) treatment on BCK (A-D) or CRT (E) immunoreactivity in the rat hippocampus**. As compared to controls, Cr or Tat-BCK treatment increased BCK immunoreactivity in the rat hippocampus. GPA treatment reduced BCK and CRT immunoreactivity in ther at hippocampus. Rectangles in panels 1 indicate the regions of panels 2 and 3. Bar = 200 (panel 1) and 25 (panels 2-4) μm.

uMtCK immunoreactivity was unaffected by Cr, GPA or tat-BCK treatment, as compared to saline or tat-protein treatment (Figures 2A-D). As compared to controls (Figures 3A), BCK immunoreactivity in Cr treated group was increased to 3.6-fold of control levels (Figures 3B, P < 0.01). However, BCK immunoreactivity in GPA treated animals was noticeably absent in the hippocampus (Figures 3C, P < 0.01). Similar to Cr treated animals, tat-BCK treatment enhanced BCK immunoreactivity in astrocytes to 2.6 fold of control levels (Figures 3D, P < 0.05). In addition, a few granule cells and interneurons showed BCK immunoreactivity following tat-BCK treatment (Figures 3D). CRT immunoreactivity was unaffected by Cr or tat-BCK treatment, as compared to saline or Tat-protein treatment (data not shown). However, CRT immunoreactivity in GPA treated animals was noticeably absent in the hippocampus (Figures 3E, P < 0.01).

### The effects of Cr, GPA and tat-BCK on PILO-induced seizure activity in the rat hippocampus

**Figure 4:**
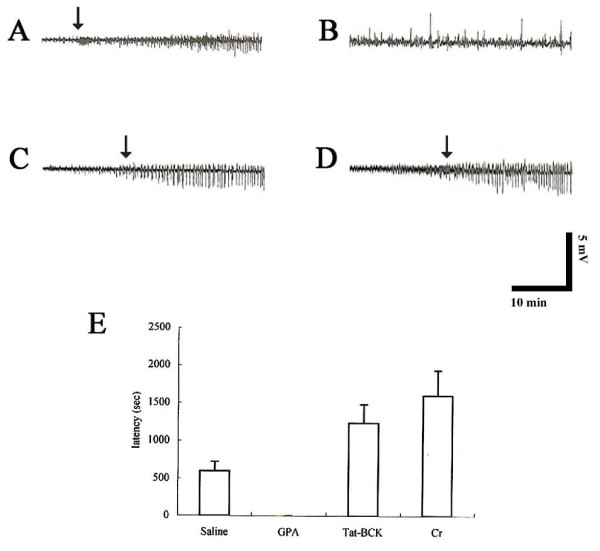
**The effect of saline (A), GPA (B), Tat-BCK (C) or Cr (D) on latency of PILO-induced seizures**. (A-D) Representative field potential recordings. (E) The histograms of pooled data showing the latency of PILO-induced seizures. GPA treatment induces spontaneous seizure activity without PILO injection. Significant differences from the controls, *P < 0.05 (paired Student's *t*-test).

In control (saline-treated) animals, PILO induces epileptiform discharges at 598 s after PILO injection (I.P.). Tat-protein treatment could not affect PILO-induced seizure activity (data not shown). However, Cr and tat-BCK treatment delays the beginning of epileptiform discharges up to 1236 s or 1598 s after PILO injection, respectively (P < 0.05). Thus, Cr and Tat-BCK treatment increased the latency of seizure onset 2- and 2.5-fold as compared with controls, respectively. GPA treatment induced spontaneous seizure activity without PILO treatment (Figures 4).

### CK and CRT expressions in the hippocampus of chronic epilepsy rat

**Figure 5:**
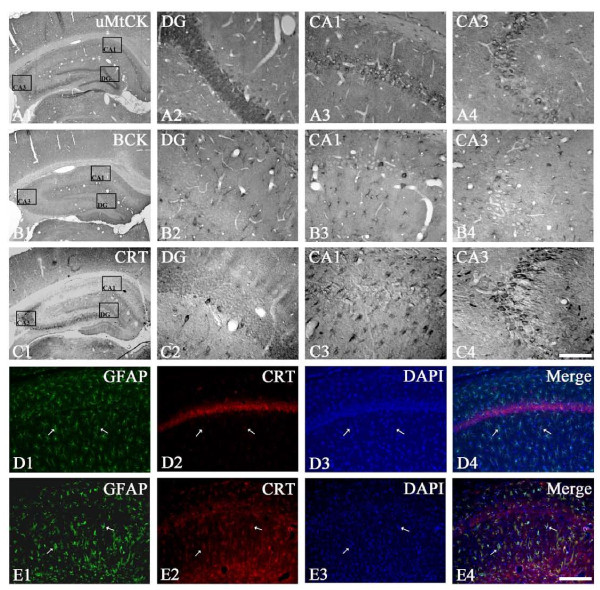
**Changes in uMtCK (A), BCK (B) and CRT (C) immunoreactivity in the hippocampus of chronic epilepsy rat**. uMtCK immunoreactivity is markedly reduced in CA1-3 pyramidal cells and hilar neurons due to massive neuronal loss. In contrast, BCK immunoreactivity is similar to that observed in control animals. CRT immunoreactivity is markedly reduced in CA3 pyramidal cells. However, CRT immunoreactivity is detected in reactive astrocytes within the hippocampus. Rectangles in panels 1 indicate the regions of panels 2 and 3. Bar = 200 (panel 1) and 25 (panels 2-4) μm. (D-E) Double immunofluoresent staining for GFAP (green) and CRT (red) in the CA1 subfield of non-SE (D) and chronic epileptic rats (E). Arrows indicate the colocalization of CRT and GFAP immunoreactivity. Bar = 50 μm.

In chronic epilepsy rats, uMtCK immunoreactivity was markedly reduced in CA1-3 pyramidal cells and hilar neurons due to massive neuronal loss (Figures 5A). In contrast, BCK immunoreactivity was similar to that observed in control animals (Figures 5B). Similar to uMtCK, CRT immunoreactivity was markedly reduced in CA3 pyramidal cells (Figures 5C). In contrast to non-SE induce animals (Figures 5D), CRT immunoreactivity was detected in reactive astrocytes within the stratum radiatum of the CA1 subfields (Figures 5E).

### The effects of Cr, GPA and Tat-BCK on chronic seizure activity

**Figure 6:**
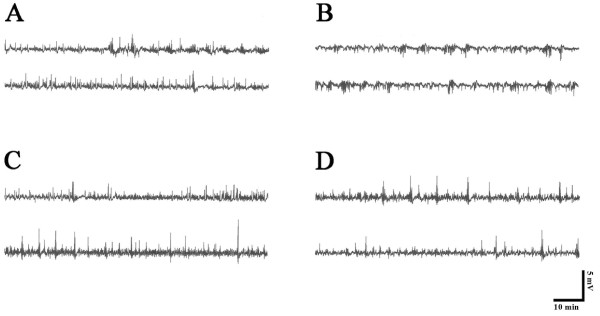
**The effect of saline (A), GPA (B), Tat-BCK (C) or Cr (D) on chronic epileptic seizure activity**. Top traces are representative EEG during baseline recording. Bottom traces are EEG after compound treatment. As compared to baseline, EEG is unaltered after Tat-protein, Cr, GPA or Tat-BCK treatment.

In chronic epilepsy rats, spontaneous synchronized spiking was detected in EEG, and long-lasting ictal discharges were observed intermittently and irregularly at least once over the 10 min recording periods. Saline- and tat-protein treatment could not affect EEG. In addition, Cr-, GPA and tat-BCK treatment could not change EEG patterns (Figures 6) or RMS values (data not shown).

## Discussion

Although multiple CK isoforms are expressed in brain [41-44], little is known regarding the localization of these isoforms of the functional role for CK in brain. Indeed, it has been reported that expressions of CKs in the brain are detected in astrocytes [45], in neurons and astrocytes [46], and in nuclei of glial cells [47]. In the present study, uMtCK immunoreactivity was observed in CA1-3 pyramidal cells, dentate granule cells and hilar neurons. In contrast, astrocytes and a small subpopulation of hilar neurons showed BCK immunoreactivity. In addition, CRT immunoreactivity was observed in dentate granule cells and CA3 pyramidal cells. These findings are agreement with a previous study demonstrating the localization of CKs and CRT in the rat hippocampus [48,49].

Seizure activity is one of the acute high-energy demand situations showing an increase in local cerebral glucose utilization [25], a higher CK rate constant and a high ATP turnover [26]. Therefore, Cr and CKs would play a role in providing a large amount of energy to be required for seizure progression. In the present study, BCK and CRT immunoreactivities were decreased following acute seizure, while uMtCK immunoreactivity was unaltered. Considering a slower chemically induced seizure development in BCK KO mice [27], these findings are simply indicated that reduced BCK immunoreactivity would be a compensatory response for seizure activity. However, the present study showed that BCK expression was mainly detected in hilar interneurons, which are vulnerable to seizure-induced insults [38]. It has been reported that PILO injection induces astroglial degeneration in the molecular layer of the dentate gyrus [34,35,50]. Furthermore, these alterations are accompanied by neuronal excitability [38,50]. Therefore, our findings reveal that reduced BCK immunoreactivity induced by PILO may result in dysfunction of hilar interneurons and astrocyte, or at least indicate decreases in their activity. The fast-spiking capability play a role in the responsiveness of inhibitory neurons [51-56]. Therefore, it is conceivable that reduced BCK expression/activity may result in a loss of fast-firing capability to allow the development of uncontrolled discharges. Furthermore, it is noteworthy that astroglial activity can change electrophysiological properties in a synaptic transmission independent manner: enhanced astroglial glutamate release, reduced glutamate reuptake, reductions of glutamine synthase and glutamate dehydrogenase, and impaired K^+ ^buffering in response to seizure activity [38,57-60]. Taken together, these reports led us to speculate on a possible enhanced seizure activity induced by reduced BCK expression/activity in hilar interneurons and astrocytes. In the present study, indeed Cr or tat-BCK treatment reduced PILO-induced acute seizure susceptibility. In addition, GPA treatment induced epileptiform discharges without PILO application. With respect to suppressing seizure activity and increasing neuronal survival by Cr (Cr-like compound) feeding [20,21], our findings suggest that reduced BCK immunoreactivity may be a practical cause of abnormal discharge rather than an indicative of a compensatory consequence of seizure activity, and that maintenance of Cr-PCr/CK circuit by BCK may play an important role in increasing acute/initial seizure threshold.

In the present study, uMtCK immunoreactivity was markedly reduced in CA1-3 pyramidal cells and hilar neurons due to massive neuronal loss. In contrast, BCK immunoreactivity was similar to that observed in control animals. Furthermore, Cr-, GPA and tat-BCK treatment could not change EEG patterns or RMS values. Vielhaber et al. [61] reported that Cr feeding has deleterious effects on pyramidal cell survival in the PILO model of temporal lobe epilepsy, since mitochondrial enzyme activities are decreased in epileptic rats and Cr feeding induces more significant decrease in mitochondrial enzyme activities. With respect to this previous study, the present data demonstrating the ineffectiveness of Cr-, GPA and tat-BCK on EEG in epileptic rats are not surprising. Although the exact biological mechanism is unclear, furthermore, the phenomenon may be a consequence from loss of mitochondria or defective oxidative phosphorylation in epileptic hippocampus [61]. Further detailed studies are needed to elucidate the roles of Cr and its metabolism in spontaneous seizure activity of chronic epileptic rats.

CRT expresses in neurons and oligodendroglia in physiological condition. Under physiological conditions, therefore Cr can cross from blood to brain through the blood-brain barrier (BBB) [15], but with a low permeability [29], partly because astrocytes lining the BBB do not express CRT [62-64]. However, Acosta et al. [65] reported the localization of CRT in perivascular astrocytes within mouse retina. Furthermore, Braissant et al. [66] have recently reported that NH_4_Cl induced CRT expression in astrocytes, including the swollen astrocytes that develop during NH_4_^+ ^exposure. Therefore, they suggested that the Cr level-dependent CNS gene regulation for CRT depends on the cell types considered and the pathological state of the brain. Similarly, the present study revealed that CRT expression was observed in reactive hypertrophic astrocytes in the hippocampi of epileptic rats, while CRT immunoreactivity was markedly reduced in CA3 pyramidal cells. Therefore, our findings support that CRT may express in astrocytes in pathological condition, although CRT expression in astrocytes is rarely detected in the brain under physiological condition. Indeed, the properties of reactive astrocyte are different from those of naive astrocyte [34,35,38,50] Taken together with preservation of BCK expression in reactive astrocytes, our findings suggest that maintenance of Cr-PCr/CK circuit in reactive astrocytes may be involved in migration, proliferation and differentiation of reactive astrocytes in epileptic hippocampus described in previous studies [34,38,50].

## Conclusion

In the present study, uMtCK immunoreactivity was markedly reduced in the hippocampi of chronic epilepsy rats as compared to control animals, while BCK immunoreactivity was similarly observed as that of controls. In addition, CRT immunoreactivity was increased in reactive astrocytes, while its immunoreactivity was significantly decreased in neurons. These findings would be simply interpreted that impaired CRT or CK functions may result in imbalance of Cr-PCr/CK circuit in chronic epilepsy animals. However, Cr-, GPA and tat-BCK treatment could not affect EEG patterns in chronic epilepsy rat. Therefore, our findings suggest that, unlike acute seizure, Cr-PCr/CK circuit may not play a role in sustaining or exacerbating spontaneous seizure activity in the epileptic hippocampus.

## Authors' contributions

DWK and SIY were involved in designing and performing all experiments. HJR, JEK, HKS, OSK, SYC and TCK helped in drafting the manuscript. HJR and JEK did immunohistochemistry and electrophysiology, acquisition of data and analyses. TA, DB, RD'H helped in behavioral studies. HKS helped in seizure studies. OSK, SYC and TCK provided continuous intellectual input, evaluation and interpretation of data. SYC and TCK conceived, designed and coordinated the project, and drafted the manuscript. All authors read and approved the final manuscript.
